# Treatment with the vascular disruptive agent OXi4503 induces an immediate and widespread epithelial to mesenchymal transition in the surviving tumor

**DOI:** 10.1002/cam4.109

**Published:** 2013-08-18

**Authors:** Theodora Fifis, Linh Nguyen, Cathy Malcontenti-Wilson, Lie Sam Chan, Patricia Luiza Nunes Costa, Jurstine Daruwalla, Mehrdad Nikfarjam, Vijayaragavan Muralidharan, Mark Waltham, Erik W Thompson, Christopher Christophi

**Affiliations:** 1 Department of Surgery, University of MelbourneAustin Health, Heidelberg, Victoria, 3084, Australia; 2 St. Vincent's Institute of Medical ResearchFitzroy, Victoria, 3065, Australia; 3 Department of Surgery, University of Melbourne, St. Vincent's HospitalFitzroy, Victoria, 3065, Australia

**Keywords:** EMT, growth factor, hypoxia, OXi4503, vascular disruptive agent, ZEB1

## Abstract

Epithelial to mesenchymal transition (EMT) is considered an important mechanism in tumor resistance to drug treatments; however, in vivo observation of this process has been limited. In this study we demonstrated an immediate and widespread EMT involving all surviving tumor cells following treatment of a mouse model of colorectal liver metastases with the vascular disruptive agent OXi4503. EMT was characterized by significant downregulation of E-cadherin, relocation and nuclear accumulation of β-catenin as well as significant upregulation of ZEB1 and vimentin. Concomitantly, significant temporal upregulation in hypoxia and the pro-angiogenic growth factors hypoxia-inducible factor 1-alpha, hepatocyte growth factor, vascular endothelial growth factor and transforming growth factor-beta were seen within the surviving tumor. The process of EMT was transient and by 5 days after treatment tumor cell reversion to epithelial morphology was evident. This reversal, termed mesenchymal to epithelial transition (MET) is a process implicated in the development of new metastases but has not been observed in vivo histologically. Similar EMT changes were observed in response to other antitumor treatments including chemotherapy, thermal ablation, and antiangiogenic treatments in our mouse colorectal metastasis model and in a murine orthotopic breast cancer model after OXi4503 treatment. These results suggest that EMT may be an early mechanism adopted by tumors in response to injury and hypoxic stress, such that inhibition of EMT in combination with other therapies could play a significant role in future cancer therapy.

Vascular disruptive treatments effectively destroy over 90% of solid tumors with minimal effects on host tissues but a viable rim of cells persists in the tumor periphery that leads to recurrence. An immediate and widespread epithelial to mesenchymal transition (EMT) occurs within the viable rim after treatment that may be responsible for this resistance to treatment. Targeting EMT in combination with vascular disruptive agents or other therapies in the clinic may improve treatment outcomes.

## Introduction

The majority of cancer-related deaths (90%) occur due to uncontrolled metastasis despite the development of wide range of systemic, regional, and local therapies. Disseminated metastases that are not amenable to surgical resection are usually treated with systemic chemotherapies. Such treatments nonspecifically target proliferating tumor cells, with cytotoxic effects on proliferating host cells and are associated with the development of drug resistance^1^. Tumor targeting approaches have recently been explored either targeting pathways and molecules that are specifically or preferentially expressed in tumors, or exploiting the differential sensitivity of the tumor vasculature to antiangiogenic drugs and vascular disruptive agents (VDAs). The clinical efficacy of these new therapies has not yet lived up to their experimental promise with the majority of patients developing eventual recurrence^2^. Drugs that specifically target existing tumor vasculature can effectively destroy more than 90% of a solid tumor^3^. A small number of resistant tumor cells survive in the tumor periphery after treatment and invariably result in recurrence. This localized resistance to VDAs has been reported in many preclinical tumor models and in clinical trials in different types of malignancies^4^. Localized resistance is likely due to significant heterogeneity within a solid tumor. This arises not only from genetic mutations accumulating by the continuous proliferation of tumor cells, but also due to differences in the local microenvironment of individual tumor cells^1^. In a recent study we demonstrated tumor cells that survive treatment with the VDA OXi4503 in the periphery have a significantly different microenvironment to the rest of the tumor^5^. These differences included vascular morphology, oxygen availability, cytokine and growth factor composition, and stromal cell association. A number of other published studies have also reported significant tumor and stromal differences between the bulk of the tumor and the invasive front (included in the periphery of our studies) and suggest these differences contribute to drug resistance^6^ and metastasis^7^,^8^. In our earlier study we demonstrated reduced proliferation and apoptosis within the viable rim compared with the rest of the tumor after vascular targeting treatment^5^. In the present study we examined molecular and morphological changes in the surviving tumor cells after treatment, which may enable them to avoid apoptosis. We identified significant temporal changes in the expression of several growth factors within the surviving tumor region, and most notably we showed a widespread but transient EMT involving all of the surviving tumor cells. EMT is known from in vitro studies to confer resistance to drug treatment and is implicated in the development of tumor resistance in vivo^9^,^10^. We also observed similar EMT changes when other antitumor treatments are used including chemotherapy, thermal ablation, and antiangiogenic treatments.

## Material and Methods

### Animals

Six- to eight-week-old male CBA mice (Laboratory Animal services, University of Adelaide, South Australia) were used in all CRC liver metastases (CRCLM) experiments. Five- to seven-week-old female BALB/c mice (WEHI, Melbourne, Australia) were used for the mouse mammary cancer experiments. Mice were maintained in standard cages with access to irradiated food and water ad libitum, and exposed to a twelve hour light/dark cycle. All procedures were implemented in accordance with the guidelines of the Austin Animal Ethics Committee.

### Experimental model of CRCLM

The primary mouse colorectal (MoCR) cancer cell line was derived from a dimethyl hydrazine (DMH)-induced primary colon carcinoma in the CBA mouse and maintained in vivo by serial passage in the flanks of CBA mice^11^. For passage and experimentation, subcutaneous tumors were teased apart, passed through a filter, treated with ethylenediaminetetraacetic acid (EDTA), and washed in phosphate buffered saline (PBS) to make a single cell suspension. Liver metastases were induced by intrasplenic injection of 5 × 10^4^ tumor cells followed by splenectomy^11^. Metastases are fully established by 21 days following tumor induction^11^.

### Experimental model of mammary cancer (4T1.2)

A mouse model of spontaneous breast cancer metastasis was used as previously described^12^. 4T1.2 cells (kindly provided by A/Prof. Robin Anderson, Peter MacCallum Cancer Centre, Melbourne, Australia) were routinely cultured in DMEM (Gibco BRL, Victoria, Australia) containing 10% fetal bovine serum (Gibco BRL, Victoria, Australia) and in a 37°C/5% CO_2_ humidified incubator. A 15 μL solution containing 1 × 10^5^ 4T1.2 cells were inoculated bilaterally into the mammary fat pad, then tumors grown for 14 days. Four animals per group were used. Tumors had a 100% take rate. Mice then received either vehicle (saline) or a single dose of OXi4503 at day 14 as described below.

### OXi4503 treatment protocol

Treatment was administered 16 days after induction of liver metastases or 14 days after breast tumor induction when tumors are well established. OXi4503, kindly donated by OXiGENE (OXiGENE® Inc. South San Francisco, CA), was freshly prepared by dissolving in 0.9% sterile saline (NaCl) while being protected from light. A single maximum tolerated dose of OXi4503, previously determined to be 100 mg/kg, was administered via intraperitoneal injection^13^. Control groups were administered an equivalent volume of sterile saline. Tissues were collected at 1 h, 24 h, and 5 days following OXi4503 treatment.

### Other treatment modalities

Tumor tissues from three other treatment modalities were examined for evidence of EMT changes. In each case tumor metastases were established as described above and a minimum of four animals per group were used. In the first treatment mice bearing liver metastases were treated with a single dose of the cytotoxic agent SMA-pirarubicin at 18 days post tumor induction. The drug dose of 250 mg/kg SMA-pirarubicin is equivalent to 100 mg free pirarubicin and was given intravenously via the tail vein in 0.2 mL saline solution^14^. Tissues from that study were collected at 3 days after treatment and stained for EMT markers. The second treatment investigated ablated tumor tissues from mice that had two selected tumor metastases ablated by laser application on day 21 post tumor induction as described previously^15^. Tissues from that study were collected at 24 h following treatment and stained for EMT markers. The third treatment investigated the effects of a single dose of sunitinib at 40 mg/kg, administered IP on day 16 post tumor induction. Tissues from that study were collected at 24 h following treatment and stained for EMT markers.

### Definition of tumor periphery

Tumor periphery in our studies consisted of the area encompassing the tumor–host interface and extending one hundred microns toward the tumor center. All the remaining tumor area was considered part of the tumor center.

### Detection of tumor hypoxia

Pimonidazole was used as a marker of tumor hypoxia. Pimonidazole hydrochloride was dissolved into 0.9% NaCl and administered intravenously to tumor-bearing mice in doses of 30 mg/kg. The livers were removed 1 h after pimonidazole administration and fixed in 10% formalin in 0.1 mol/L phosphate buffer, pH 7.2. Hypoxic tumor regions were detected immunohistochemically by established techniques^16^.

### Assessment of epithelial to mesenchymal transition

The main indicators of epithelial to mesenchymal transition (EMT) are downregulation of E-cadherin (cell junction protein), nuclear accumulation of β-catenin (cell junction protein), upregulation of the mesenchymal marker vimentin, and upregulation of transcriptional drivers of EMT such as ZEB1^17^,^18^. Changes in these markers were assessed using immunohistochemistry and immunoblot.

### Immunohistochemistry

Table S1 presents a list of antibody concentrations and assay conditions used. Formalin-fixed paraffin tissue sections (4 μm) were used with an indirect peroxidase labeling technique (Envision Plus, DAKO, Campbellfield, Australia). Following deparaffinization and rehydration, endogenous peroxidase activity was blocked with 3% H_2_O_2_ and nonspecific binding inhibited with 10% normal goat serum (01-6201 Zymed Laboratories, San Francisco, CA). Epitope retrieval was conducted as per Table S1. Sections were incubated with primary antibodies or the respective nonimmune antibody isotypes overnight at 4°C. Sections treated with the rat antibodies were subsequently treated with a rabbit anti-rat IgG linker antibody before treatment with a polymer-based detection kit containing goat anti-rabbit immunoglobulins (IgG) linked to horseradish peroxidase (HRP) (Envision Plus, Dako). Each incubation step was followed by 2- to 5- min washes with PBS + 0.05% Tween 20. Positive staining was visualized using diaminobenzidine (DAB) as a substrate. Between 75 and 120 tumors were assessed for each time point/treatment group. Each group consisted of a minimum of five animals and up to a maximum of 10. Each animal had several liver metastases (discrete tumors) of various sizes. Liver sections were taken to include the entire liver in the analysis and each section contained from one to several tumors. Individual tumors were often represented in more than one sections. Images of stained tumors were captured using a digital light microscope (Nikon Coolscope®, Nikon Corporation, Japan) at between 10× and 400× magnification. The images of tumor fields were captured to be representative of the entire tumor periphery, using a raster pattern which allowed for fields captured to be random and not overlap^19^. Between 10 and 30 fields per tumor were assessed. The images were analyzed using Image-Pro plus (Version 5, Media Cybernetics, Perth Australia). Only areas of viable tumor were considered in the analysis. Changes in hypoxia and the antigens AT1R, vascular endothelial growth factor (VEGF), hypoxia-inducible factor 1-alpha (HIF-1α), E-cadherin, Vimentin, β-catenin, and ZEB1 were assessed by microscopy and representative images are presented. Changes in expression wherever possible were evaluated using a semi-quantitative scoring system. Areas of interest were identified using a light microscope (Olympus BH2, Tokyo, Japan) at a magnification of 125×. Scoring criteria was used to estimate the amount and intensity of staining seen in each sample. The grading system used was as follows: 0: no staining 1: faint staining; 2: small amount or weak staining; 3: moderate staining; 4: abundant or strong staining; 5: Abundant or very strong staining. Means for each group were determined using the individual average scores from each animal in the group. Scoring researchers were blinded in regard to the experimental group.

### Preparation of liver and tumor tissue lysates

Collected tissues from control and treated animals were snap-frozen in liquid nitrogen to be used for protein preparations. Each sample contained several individual tumor metastases from the same animal or several liver sections from different areas of the liver for each animal. The frozen tissues were weighed into microcentrifuge tubes and kept on ice. Cold lysis buffer (50 mmol/L hydroxyethyl piperazineethanesulfonic acid (HEPES), 150 mmol/L NaCl, 10 mmol/L EDTA, 10 mmol/L Na_4_P_2_O_7_, 10 mmol/L NaF, 2 mmol/L Na_3_VO_4_, 0.5 mmol/L phenylmethylsulfonyl fluoride, 20 μmol/L Leupeptine, 10 μg/mL Aprotinin, and 1% Triton X-100) was added to each tissue at a ratio of 500 μL/mg tissue weight. The tissues were homogenized using an Ultra Turrax T25 homogeniser (John Morris Scientific Pty Ltd., Sydney, Australia) in three ten second bursts while the sample was kept on ice. The homogenates were then sonicated with three 10 second bursts using a Branson Sonifier 250 (Branson Ulrasonics Co., Danbury, CT) at 30 Hz constant output, while the sample was kept on ice. The sonicates were centrifuged at 17,000 g at 4°C for 15 min and the supernatant (lysate) was collected, aliquoted, and kept frozen at −80°C until used. Protein concentrations were determined by the Bradford reagent (Sigma).

### Immunoblot

Pooled tumor lysates representing equal amounts of protein from each animal in the group (*n* = 4 per group) were resolved by sodium dodecyl sulfate polyacrylamide gel electrophoresis and proteins were detected with antibodies against E-cadherin and GAPDH (Genesearch, Melbourne, Australia). The bound antibodies were visualized using ECL reagents (GE Healthcare, Buckinghamshire, U.K.). Relative changes in E-cadherin concentration was determined by normalization using GAPDH as internal standard.

### Enzyme linked immuno sorbent assay

The levels of the growth factors VEGF, hepatocyte growth factor (HGF), and TGF-β1 in tissue extracts were determined by a sandwich enzyme linked immuno sorbent assay (ELISA) using commercially available kits (mouse Duosets, VEGF Cat No: DY493 and TGF-β1 DY1679, R&D Systems, Minneapolis MN) according to the manufacturer's instructions with minor modifications. In brief 96-well microplates (Nunc Immuno Plate maxisorb, In Vitro Technologies, Victoria, Australia) were coated with 50 μL per well of suitably diluted capture antibody and incubated overnight at 4°C. Following blocking with reagent diluent, the test samples and standards were applied at appropriate concentrations. Transforming growth factor-beta (TGF-β) samples were activated using 2.5 N Acetic Acid/10 mol/L and neutralized by 2.7 N NaOH/1.0 mol/L HEPES before being applied onto the ELISA plate. After 2 h incubation, the biotinylated detection antibody was added followed by Streptavidin-HRP. The plate was washed before each successive step with 0.05% Tween 20 in PBS. Substrate (H_2_O_2_ and Tetramethylbenzidine, R&D Systems Catalog number DY994) was added and developed for 20 min at room temperature, followed by the addition of 25 μL of stop solution (2N H_2_SO_4_). The optical density was determined immediately using a BioRad microplate reader (Benchmark Plus Microplate Spectrophotometer System, Bio-Rad Laboratories, Hercules, CA) set at dual wavelengths of 450 and 540 nm. The 540 nm reading was subtracted from the 450 nm reading. Using a computer generated four parameter logistic curve fit from the standard concentrations (4-PL), the unknown sample concentrations were directly calculated by the spectrophotometer software.

### Statistical analysis

Quantitative data are represented as the mean ± standard error of the mean. Statistical analysis was conducted using SPSS (Statistical Package for the Social Sciences,® version 10, Chicago, IL) with normality testing and use of both parametric and nonparametric analytical tests as appropriate. All statistical tests were two sided and a *P*-value of 0.05 or less was considered statistically significant.

## Results

### Histological changes following Oxi4503 treatment

**Figure 1:**
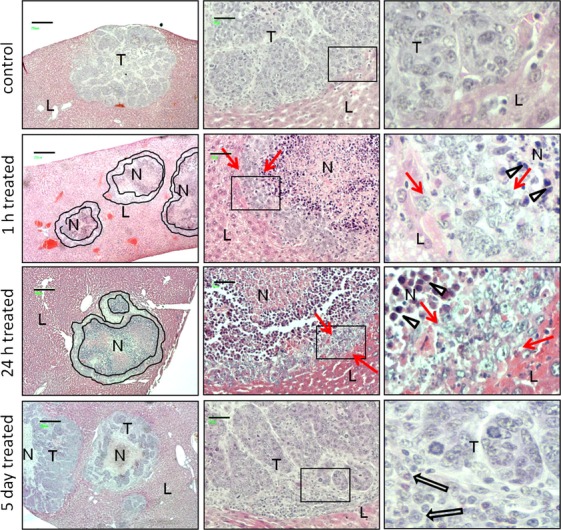
Hematoxylin and eosin staining of CRCLM tumors following OXi4503 treatment. Mice with liver metastases were treated with a single IP dose of OXi4503 (100 mg/kg) at 16 days post tumor induction. Tissues were collected at indicated times following OXi4503 treatment. First column, black bar = 250 μm, second column, black bar = 50 μm, third column = magnified inset of second column. L, liver; T, tumor; N, treatment induced necrosis. Double black lines in the second and third panels first column outline the viable tumor rim at 1 and 24 h posttreatment. Single red arrows in second and third panels of columns 2 and 3 indicate the viable tumor rim and arrowheads indicate apoptotic cells. Double arrows in the 5 day treated magnified inset indicate single tumor cells not yet organized into nodules.

One hour after Oxi4503 treatment, widespread apoptosis and necrosis occurred in the center of the tumor due to vessel occlusion. This can be seen by the fragmenting and pyknotic cell nuclei staining indicating apoptosis and often total absence of nuclear staining indicating necrosis in hematoxylin and eosin (H&E) staining (Fig. 1, 1 h treated). Apoptosis was also evident by extensive caspase 3 staining (Fig. S1, 1 h treated). Intact viable tumor was present only in a thin rim at the tumor periphery as can be seen by their normal nuclear morphology. (Fig. 1, 1 h treated). Even within the viable rim several tumor cells were caspase positive indicating that not all tumor cells in this area survive (Fig. S1, 1 h treated). Twenty-four hours after treatment tumor cells in the viable tumor rim appeared disorganized and less dense than in the control or at 1 h time point (Fig. 1, 24 h treated); however, also fewer cells were caspase positive (Fig. S1, 24 h treated). By day five tumor growth had resumed with tumor cells growing into the necrotic center. The majority of tumor cells were organized into nodules; however, there were many single tumor cells not organized (Fig. 1, 5 day treated, third panel, double arrows), and large infiltration of host cells were seen into the tumor especially in the tumor–host interface (Fig. 1, 5 day treated, middle panel). Some apoptotic cells were seen among the new tumor growth mainly confined toward the necrotic center (Fig. S1, 5 day treated).

### OXi4503 treatment induces acute hypoxia and upregulation of pro-angiogenic growth factors in the surviving viable tumor

**Figure 2:**
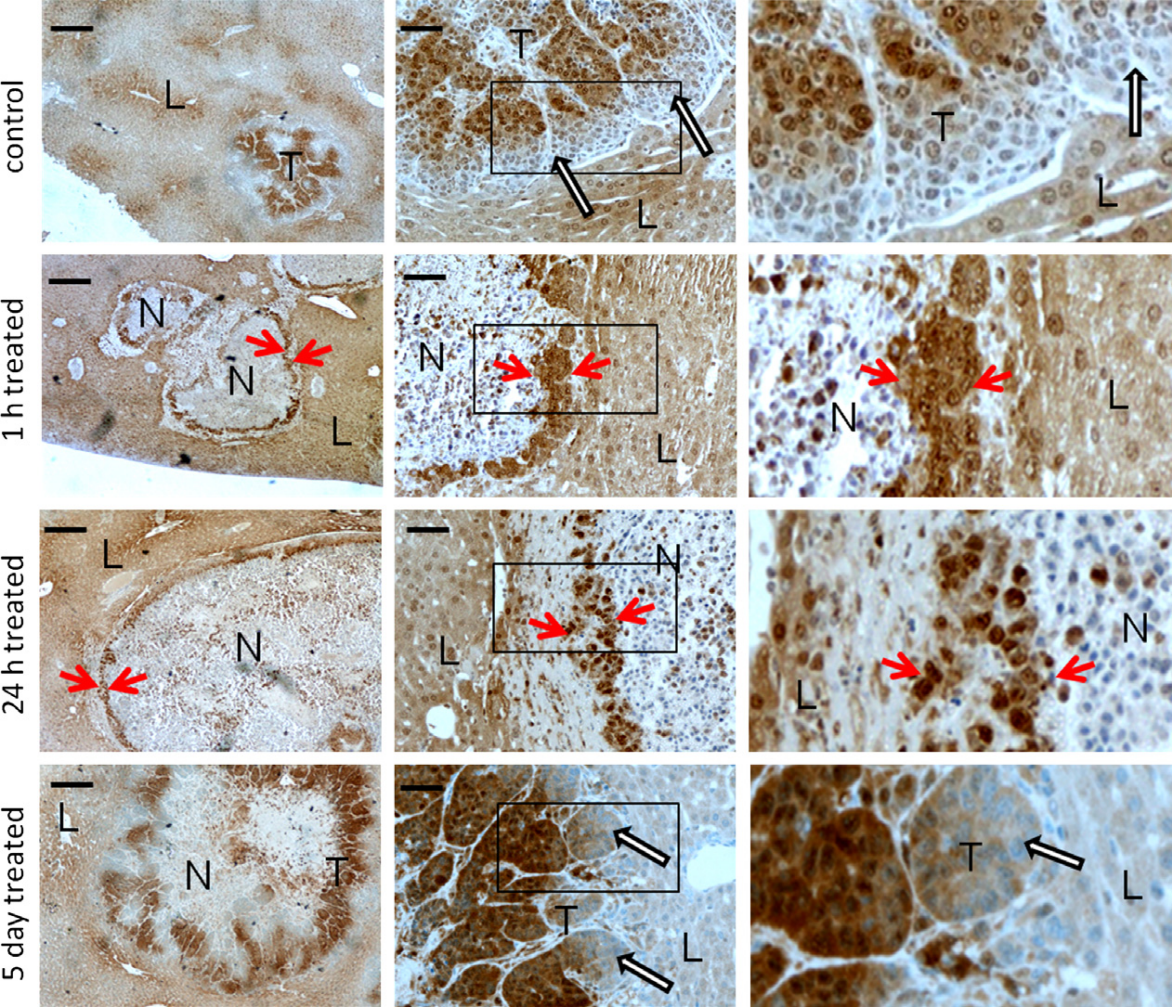
Changes in hypoxia in CRCLM tumors following OXi4503 treatment. Mice with liver metastases were treated with a single IP dose of OXi4503 (100 mg/kg) at 16 days post tumor induction. Tissues were collected at indicated times following OXi4503 treatment. Hypoxia was detected by Pimonidazole staining (brown staining indicating positive for hypoxia). First column, black bar = 250 μm, second column black bar = 50 μm, third column = magnified inset of second column. L, liver; T, tumor; N, treatment induced necrosis. Double arrows indicate tumor at the tumor–host interface in the control and the regenerating tumor at 5 days after treatment showing minimal hypoxia. Single red arrows in all panels of second and third row indicate tumor cells within the viable rim at 1 and 24 h staining strongly for hypoxia.

**Figure 3:**
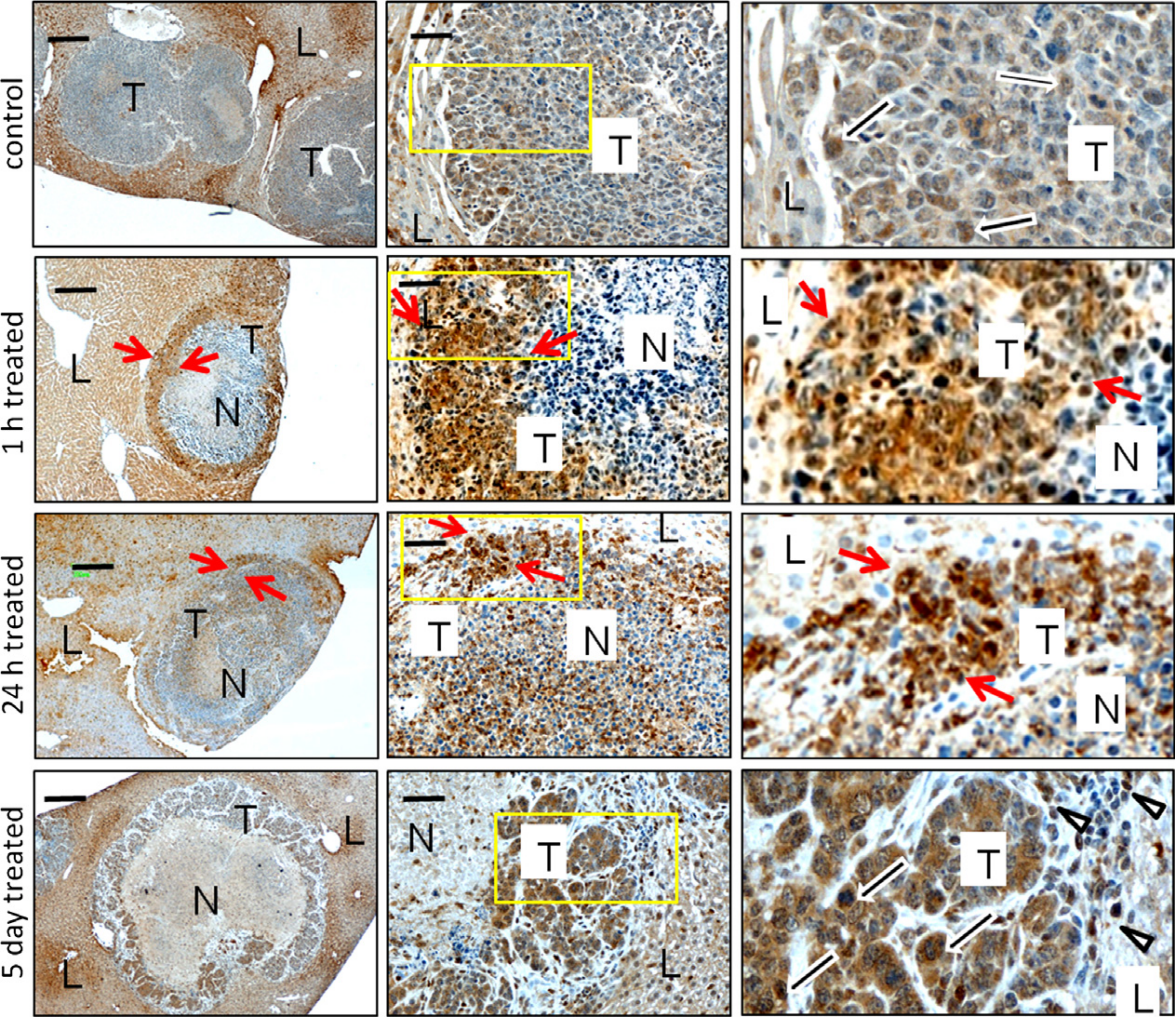
Changes in HIF 1α expression levels in CRCLM tumors following OXi4503 treatment. Mice with liver metastases were treated with a single IP dose of OXi4503 (100 mg/kg) at 16 days post tumor induction. Tissues were collected at indicated times following OXi4503 treatment. Expression of HIF 1α (brown staining) was detected using anti-HIF 1a antibodies. First column, black bar = 200 μm, second column black bar = 50 μm, third column = magnified inset of second column. L, liver; T, tumor; N, treatment induced necrosis. Double arrows indicate occasional tumor cells showing strong nuclear staining. Single red arrows in all panels of second and third row indicate that tumor cells within the viable rim at 1 and 24 h show strong cytoplasmic and nuclear staining for HIF 1α. Arrowheads indicate infiltrating cells with strong HIF 1α staining.

In control animals tumor tissue at the tumor–host interface was normoxic (Fig. 2, control, arrows) while intense hypoxia is seen in central parts of the tumor especially those distant from blood vessels. Following treatment the viable tumor rim displayed acute increase in hypoxia compared with untreated control (Fig. 2, 1 h treated, arrows). The same pattern is seen at 24 h after treatment (Fig. 2, 24 h treated). Five days after treatment the viable rim had regrown significantly and the hypoxia pattern resembled that of the untreated control and was mainly seen within regions distant from vasculature and close to necrotic areas (Fig. 2, 5 day treated). Levels of HIF-1α, known to be stabilized by hypoxia, were also investigated. In untreated tumor HIF-1α was expressed at low levels in the cytoplasm and nucleus of most tumor cells but was strongly expressed in the nucleus of a proportion of cells interspersed throughout the tumor (Fig. 3, control arrows). Following OXi4503 treatment there was an increase in HIF-1α staining in the cytoplasm and nucleus of almost all the surviving tumor cells (Fig. 3, 1 h and 24 h treated). HIF-1α levels remained high mainly in the tumor cytoplasm at day five despite reduction in hypoxia staining (Fig. 3, 5 day treated). A proportion of tumor cells displayed nuclear HIF-1α expression (Fig. 3, 5 day treated double arrows) and many HIF-1α positive infiltrating cells were seen in the tumor periphery (Fig. 3, 5 day treated, arrowheads).

**Figure 4:**
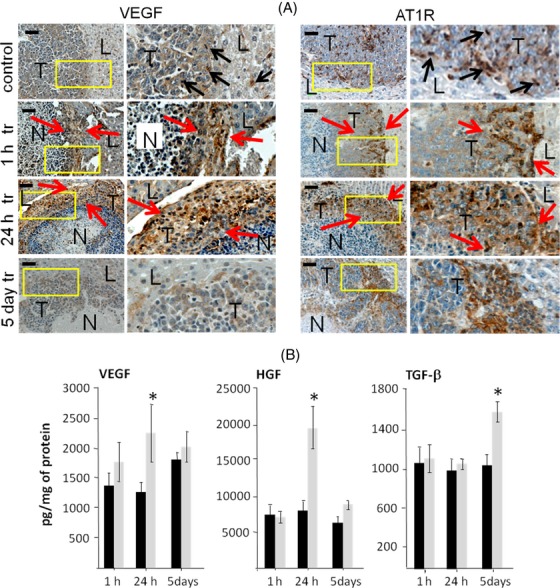
Temporal changes in proangiogenic growth factor/receptor levels in tumors following OXi4503 treatment. Mice with liver metastases were treated with a single IP dose of OXi4503 (100 mg/kg) at 16 days post tumor induction. (A) Temporal changes in VEGF and AT1R expression following OXi4503 treatment determined by immunohistochemistry. Tissues were collected at indicated times following OXi4503 treatment. Expression (brown staining) was detected using the appropriate antibodies. Black bar = 50 μm, second column and fourth column panels = magnified inset of second column and third column panels. L, liver; T, tumor; N, treatment induced necrosis. Double arrows indicate occasional tumor cells showing strong nuclear staining. Single red arrows in all panels of second and third row indicate that tumor cells within the viable rim at 1 and 24 h show increased VEGF and AT1R staining. Black arrows in second and fourth panels in the control tumors indicate infiltrating cells expressing VEGF and AT1R. (B) Temporal changes in VEGF, HGF, and TGF-β determined by ELISA. Tumor tissues were collected at indicated times following OXi4503 treatment. Growth factor concentration was determined in tumor lysates by capture ELISA assays using commercially available kits (R&D Duo Kits) ▪, control tumor bearing mice; □, Oxi4503-treated tumor-bearing mice. The data are mean values ± SEM (*n* = 10) VEGF, OXi4503 treated at 24 h *P < 0.05 compared with untreated control, HGF, OXi4503 treated at 24 h *P < 0.0009 compared with untreated control. TGF-β, OXi4503 treated at 5 days following *P < 0.0009 compared with untreated control.

In a previous study, we found upregulated expression of VEGF, ATR1, and TGF-β preferentially associated with the tumor periphery^5^. In the current study we found VEGF and ATR1 expressed mainly in the nucleus and cytoplasm of infiltrating cells and in the cytoplasm of some tumor cells in the periphery (Fig. 4A, control and Fig. S2, contol). Following OXi4503 treatment VEGF staining was further upregulated within the viable tumor and was mainly located in the tumor cytoplasm and the cytoplasm and nucleus of infiltrating cells (Fig. 4A, VEGF 1 h and 24 h treated and Fig. S2). VEGF staining returned to control levels by day five in the regrowing tumor (Fig. 4A, VEGF, 5 day treated and Fig. S2, VEGF 5 day treated). Similarly AT1R staining increased at 1 h after treatment but by day five it had returned to baseline levels (Fig. 4A and S2).

Temporal local and systemic changes in HGF, TGF-β, and VEGF in response to OXi4503 treatment were also evaluated by ELISA assays. Following treatment the concentration of HGF and VEGF significantly increased in the tumor lysates at 24 h and returned to base levels by day 5 (Fig. 4B) confirming our immunohistochemistry results for VEGF. In contrast TGF-β increased significantly only at day 5 (Fig. 4B). In addition, there was parenchymal and systemic modulation of these growth factors. VEGF levels increased significantly at 24 h in the serum and showed an increasing trend in the liver parenchyma (Fig. S3, column 3). In contrast HGF and TGF-β levels decreased in the serum following treatment; HGF showed significant decrease at 5 days and TGF-β at 24 h and 5 days (Fig. S3, row 2). HGF levels did not change in liver lysates while TGF-β significantly decreased at 24 h (Fig. S3). These results demonstrate that OXi4503 treatment exerts differential effects on local and systemic cytokine and growth factor concentration, and this modulation may contribute to tumor resistance.

### OXi 4503 treatment induces changes in EMT markers in the surviving viable tumor

**Figure 5:**
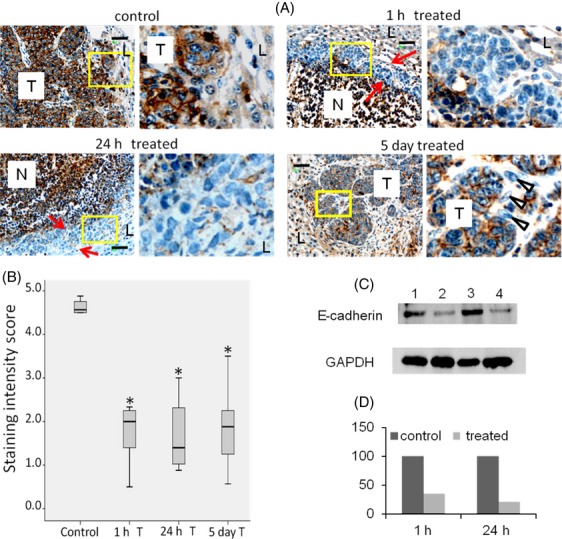
Reduction in E-cadherin following OXi4503 treatment. Mice with liver metastases were treated with a single IP dose of OXi4503 (100 mg/kg) at 16 days post tumor induction. Tissues were collected at 1, 24 h, and 5 days following Oxi4503 treatment. (A) Formalin-fixed tumor sections were stained with antibodies to E-cadherin. Positive expression is detected by the brown staining. L, liver; T, tumor; N, treatment induced necrosis. Red arrows indicate area of the viable tumor rim; Arrowheads indicate unattached tumor cells that do not express E-cadherin. Black bar = 50 μm. Panels in columns two and four represent magnification of corresponding insets in columns one and three. (B) Quantification of E-cadherin using an intensity scoring system. Untreated control versus OXi4503 treated at all time points, *P < 0.001. (C) Western blot of pooled tumor lysates (*n* = 4 per group) stained for E-cadherin, Lanes: (1) 1 h control; (2) 1 h Oxi4503 treated; (3) 24 h control; (4) 24 h Oxi4503 treated. (D) Normalization of E-cadherin concentration using GAPDH as internal standard.

The temporal increase in angiogenic growth factors led us to investigate changes in EMT markers as many studies suggested that growth factors including HIF-1α, TGF-β, VEGF, and HGF are responsible for EMT changes giving rise to invasive and metastatic tumors^20^–^22^. Downregulation of E-cadherin is regarded as the hallmark of EMT so we initially investigated changes in E-cadherin following treatment. We determined that E-cadherin was highly expressed in the untreated control tumors giving the characteristic cobblestone appearance (Fig. 5A, control and Fig. S4, control). Surprisingly, within 1 h of treatment, E-cadherin was dramatically downregulated within the entire viable tumor rim (Fig. 5A, 1 h treated and Fig. S4, 1 h treated). This downregulation persisted for 24 h posttreatment (Fig. 5A, 24 h treated and Fig. S4, 24 h treated). At this time point the tumor cell boundaries appeared disorganized and the cells had adopted a less rounded morphology. While E-cadherin staining increased again by day five, overall it was significantly lower than the control (Fig. 5A, 5 day treated and Fig. S4, 5 day treated). At day five the majority of tumor cells appeared organized in clusters but also several detached tumor cells were clearly visible (Fig. 5A, 5 day treated, inset arrowheads). Quantification of the E-cadherin changes showed significant differences between controls and OXi4503 treated groups at all three time points after treatment (Fig. 5B). E-cadherin changes were also confirmed using Western blotting of tumor lysates (Fig. 5C and D). The results clearly demonstrate decreased E-cadherin concentration following treatment, thus verifying the immunohistochemistry results.

**Figure 6:**
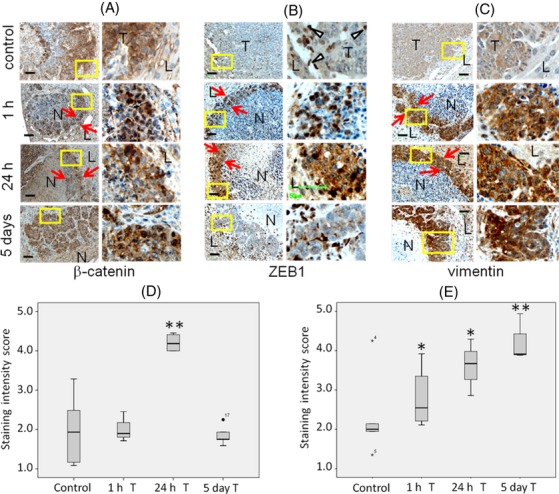
Changes in EMT markers following OXi4503 treatment of tumor metastases. Mice with liver metastases were treated with a single IP dose of OXi4503 (100 mg/kg) at 16 days post tumor induction. Tissues were collected at 1, 24 h, and 5 days following OXi4503 treatment. Formalin-fixed control and treated tumor sections were stained with (A) antibodies to β-catenin, (B) antibodies to ZEB1, and (C) antibodies to vimentin. Positive expression is detected by the brown staining. Red arrows indicate area of the viable tumor rim; arrowheads in B control panel indicate host infiltrating cells staining strongly positive for ZEB1. Black bar = 50 μm. Panels in second column of A–C depict respective magnified insets. (D and E) Quantification of ZEB1 and vimentin, respectively, using an intensity scoring system. *P < 0.05, **P < 0.015.

We next investigated changes in β-catenin as this protein combines with E-cadherin to stabilize the cell junctions. In the control tumors β-catenin displayed similar distribution to E-cadherin; staining localized at the cell junctions giving a cobblestone appearance (Fig. 6A, β-catenin control and Fig. S4). Within 1 h following treatment, redistribution of β-catenin was seen away from the adherens junctions and accumulation in the cytoplasm and nucleus (Fig. 6A, β-catenin 1 h treated and Fig. S4). Cytoplasmic and nuclear localization of β-catenin persisted at the next two time points and the nuclear accumulation became more intense at day five (Fig. 6A, β-catenin 5 day treated and Fig. S4). Changes in the ZEB1 transcription factor an E-cadherin repressor were also investigated. ZEB1 was found to be expressed in the untreated control tumors by infiltrating cells that accumulate at the tumor–host interface and along major vessels (Fig. 6B, ZEB1 control and Fig. S4). Based on their morphological appearance, these cells closely resemble fibroblasts (Fig. 6B, ZEB1 control, inset arrowheads). The occasional tumor cell also displayed positive staining. Following OXi4503 treatment at 1 h there was nuclear staining in the majority of viable cells in the tumor rim (Fig. 4B, ZEB1 1 h treated and Fig. S4). The staining increased and became predominantly cytoplasmic at 24 h after treatment. All surviving tumor cells expressed cytoplasmic ZEB1 at this time point (Fig. 6B, ZEB1 24 h treated). By day 5 posttreatment, however, only weak ZEB1 staining remained in the tumor cytoplasm and the staining pattern resembled the control with only infiltrating stroma cells displaying strong nuclear staining (Fig. 6B, ZEB1 5 day treated and Fig. S4).

Changes in vimentin staining were also examined. In the untreated control tumor there was low expression of vimentin protein (Fig. 6C, vimentin control and Fig. S4). Within an hour of treatment strong upregulation of vimentin staining was seen within the viable tumor rim with both cytoplasmic and nuclear localization clearly prominent (Fig. 6C, vimentin 1 h treated). The vimentin upregulation persisted at the next two time points with levels significantly higher than the control (Fig. 6C, vimentin 24 h and 5 day treated and Fig. S4). At day 5, the staining intensity of vimentin was stronger in the tumor cells repopulating the necrotic center of the tumor and weaker toward the tumor–host interface (Fig. 6C vimentin 5 day treated). The changes in staining were quantified for ZEB1 and vimentin expression and as indicated in the graphs (Fig. 6D and E) they were significantly higher compared with untreated controls at 24 h and 5 day time points, respectively. Taken together the results demonstrate that OXi4503 treatment induces very rapid and widespread EMT involving practically all surviving tumor cells. Furthermore, the EMT changes were transient; by day five following treatment the regrowing tumor appeared to be undergoing a mesenchymal to epithelial transition (MET) in the process of reverting to epithelial morphology as judged by the nodule arrangement, E-cadherin association with the adherens junctions and the ZEB1 downregulation. The process, however, was not totally completed as the predominant nuclear expression of β-catenin and the persistent high levels of vimentin suggest.

### Chemotherapy and other treatment modalities of liver metastases induce EMT in residual tumor

**Figure 7:**
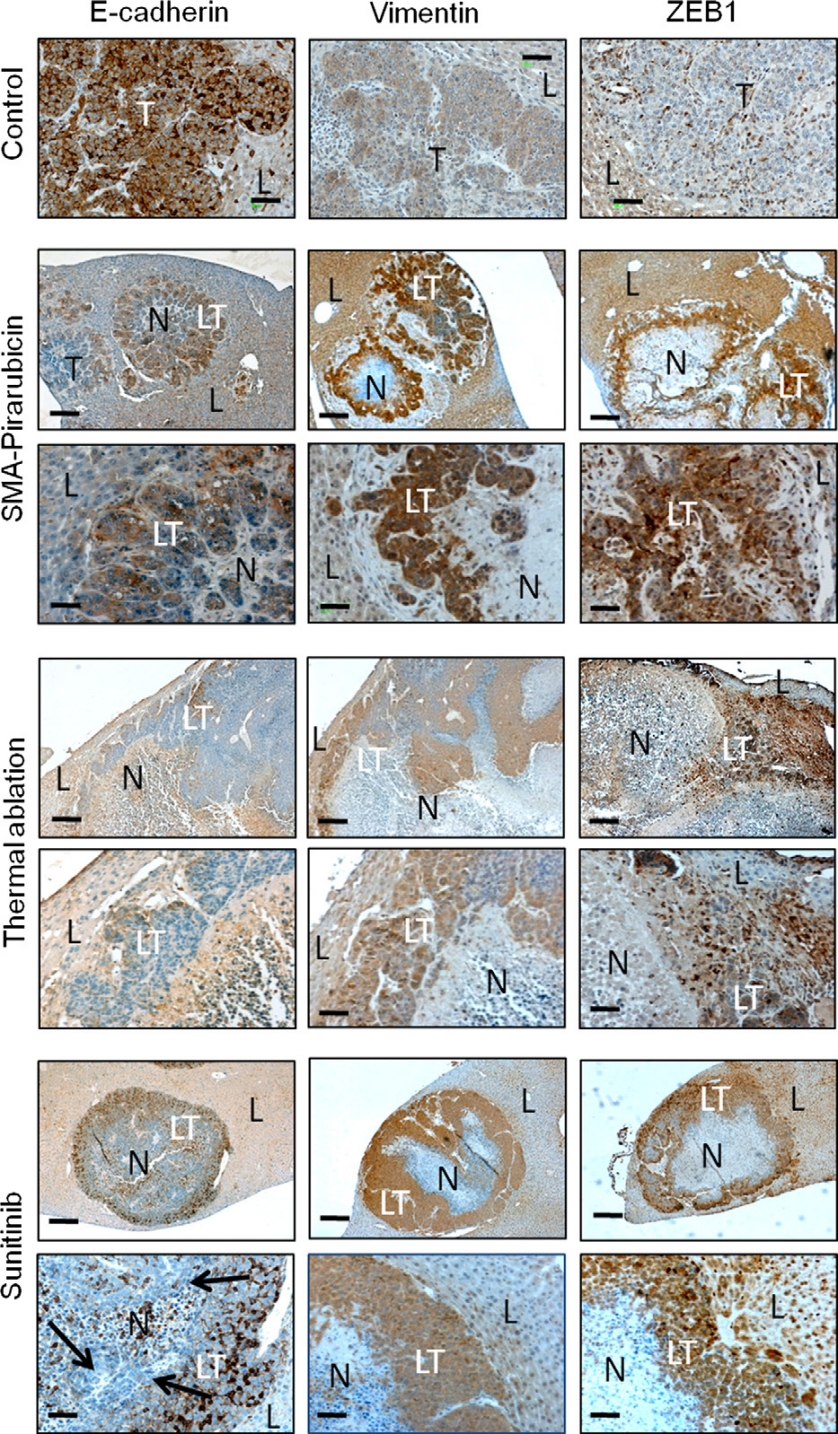
Changes in EMT markers following different treatment of CRLM tumor metastases. Formalin-fixed control and treated tumor sections were stained with antibodies to E-cadherin, vimentin, and ZEB1. Positive expression is detected by the brown staining. L, liver; T, tumor; N, necrosis resulting from treatment; LT, live tumor remaining after treatment. Low magnification scale bar = 250 μm, high magnification scale bar = 50 μm. Control tissues were collected on day 21 post tumor induction. For the SMA-pirarubicin treatment mice with liver metastases were treated with a single IP dose of SMA-pirarubicin at 18 days post tumor induction^14^. Tissues were collected 3 days following treatment. For thermal ablation mice with liver metastases had two selected tumors thermally ablated using laser treatment on day 21 post tumor induction. Tissues were collected 24 h after treatment^15^. For the sunitinib treatment mice with liver metastases were treated with a single IP dose of sunitinib at 16 days post tumor induction. Tissues were collected 24 h after treatment. Arrows in the high magnification panel of sunitinib treatment indicate reduction in E-cadherin in tumor cells adjacent to necrotic areas.

Based on the findings with OXi4503 treatment in this study we hypothesized that similar morphological changes occur in other tumor treatments, we therefore examined archival tumor tissues from previous studies for evidence of EMT induction. In a previous study we reported significant tumor damage and acute hypoxia within residual tumors after treatment with the cytotoxic agent SMA-pirarubicin^14^. Tissues from that study, collected at 3 days after a single SMA-pirarubicin treatment, were stained for EMT markers. The results showed a large decrease in E-cadherin staining and a reciprocal strong increase in the staining of ZEB1 and vimentin compared with control tumor (Fig. 7, control and SMA-pirarubicin treated). The second treatment examined was an earlier study of tumor thermal ablation by laser application. This is a localized treatment of individual tumors and results in extensive immediate tumor necrosis at the ablation site^15^. Staining of tissues collected 24 h after treatment displayed extensive decrease in E-cadherin and increase in ZEB1 and vimentin in surviving tumor adjacent to the thermal injury compared with control (Fig. 7, control and thermal ablation treated). The third treatment we investigated was the antiangiogenic agent sunitinib. This agent is used to inhibit the formation of new blood vessels and it is also known to induce the regression of microcapillaries. It is normally administered continuously, but we examined EMT changes at 24 h after a single dose. In our model this treatment results in central tumor necrosis with viable tumor persisting in the outer tumor regions (Fig. 7, sunitinib treated). Staining for EMT markers revealed a decrease in E-cadherin especially in areas adjacent to necrotic regions and increase in ZEB1 and vimentin compared with control tumor (Fig. 7, control and sunitinib treated). The sunitinib single treatment is a milder treatment compared with OXi4503 as judged by the surviving tumor mass and interestingly while vimentin and ZEB1 are upregulated throughout the remaining live tumor, E-cadherin is only downregulated near the necrotic areas resulting from the treatment (Fig. 7, control and sunitinib treated). Taken together these results indicate that EMT is a general protective mechanism adopted by MoCR tumors in response to treatment stress.

### OXi4503 treatment induces EMT changes in a murine breast cancer model

**Figure 8:**
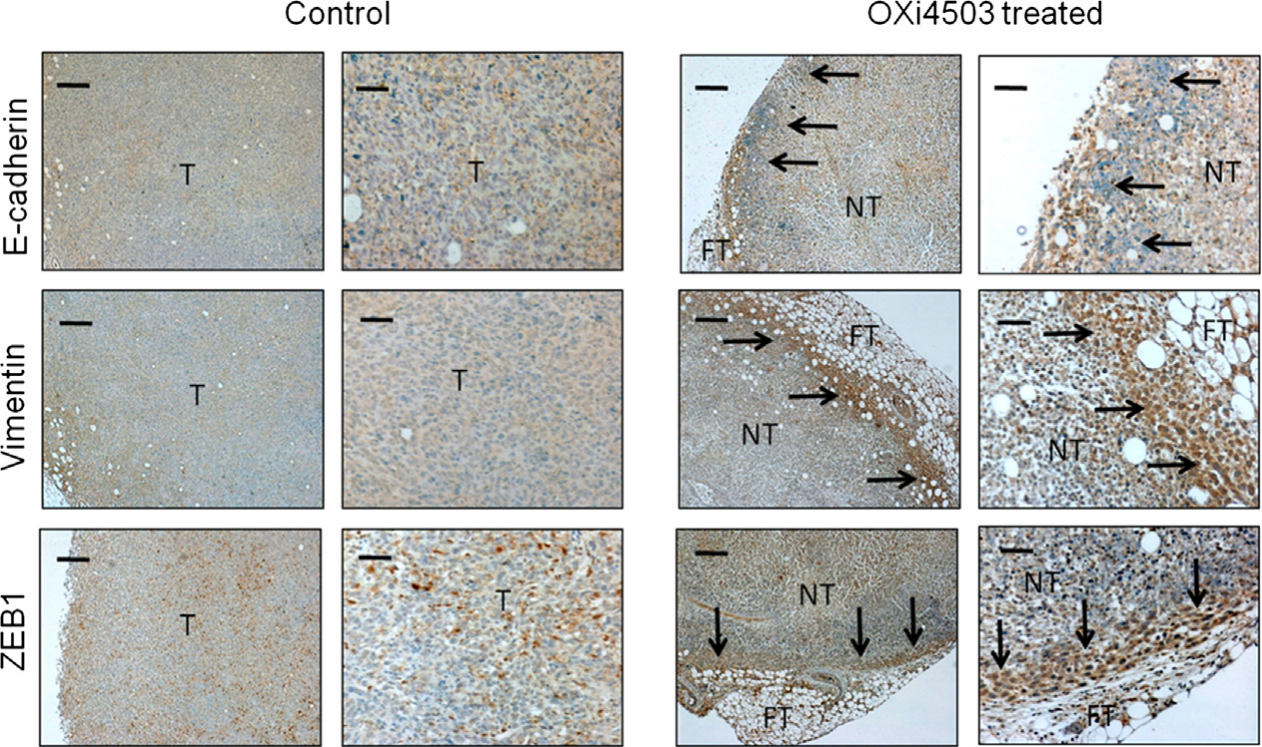
Changes in EMT markers following OXi4503 treatment in orthograft of murine breast cancer. Mice with 4T1.2 breast tumors were treated with a single IP dose of OXi4503 at 14 days post tumor induction. Tissues were collected 24 h following treatment. Formalin-fixed control and treated tumor sections were stained with antibodies to E-cadherin, vimentin, and ZEB1. Positive expression is detected by the brown staining. T, tumor; NT, necrotic tumor resulting from treatment; FT, fatty tissue. Arrows in the treated group indicate live tumor remaining after treatment. Low magnification scale bar = 250 μm, high magnification scale bar bar = 50 μm.

Epithelial to mesenchymal transition markers were also investigated in a murine breast cancer model produced by injection of a murine breast cancer cell line into the mammary fat pads of syngeneic mice. This cell line has more mesenchymal characteristics – E-cadherin is expressed at low levels and the characteristic cobblestone appearance of solid epithelial tumors is less evident. ZEB1 is expressed by infiltrating cells and some tumor cells and vimentin is also expressed at low levels (Fig. 8, control). OXi4503 treatment was administered as for the colorectal liver metastasis model without further optimization. At the dose tested, the treatment resulted in extensive tumor damage with only small patches of intact tumor surviving at the tumor periphery adjacent to host fat tissue. There was not a continuous rim of live tumor as seen in the CRCLM tumors indicating that this particular tumor is very sensitive to OXi4503. EMT changes further increased the mesenchymal characteristics of this tumor as discerned by the decrease in E-cadherin and the increase in ZEB1 and vimentin (Fig. 8). Overall these results suggest that EMT may be broadly adopted by tumors under stress to escape apoptosis.

## Discussion

Tumor treatment with VDA results in extensive tumor killing but leaves a characteristic rim of live tumor in the periphery which results in tumor recurrence^3^,^4^. In an earlier study we demonstrated significant molecular and morphological differences between the tumor periphery and the rest of the tumor that could account for the tumor resistance to VDAs in this region^5^. In the present study we investigated tumor and host responses, following OXi4503 treatment, which may further contribute to tumor survival. We demonstrated immediate increases in hypoxia and HIF-1α levels. Hypoxia stabilizes the HIF-1α protein resulting in the activation of over 100 genes. Many of these genes support angiogenesis while others are responsible for the modification of extracellular matrix, the influx of supportive cells, cytoprotection, tolerance to low oxygen levels, or promote EMT^20^. We also demonstrated temporal local and systemic changes in growth factors (HGF, VEGF, and TGF-β) that are also known to be pro-angiogenic and support tumor growth^21^. Some of these growth factors were shown in other studies to be induced by hypoxia and HIF-1α^22^,^20^. HGF and VEGF increased significantly within 24 h of treatment. Both of these factors, in addition to their angiogenic contribution, have been shown in other settings to be cytoprotective in tissue injury and cytotoxic drug treatments^23^. TGF-β did not increase significantly within the tumor until day five. Increases in growth factors and cytokines (VEGF, granulocyte-colony stimulating factor and stromal cell-derived factor 1) were also reported following VDA treatment in other tumor models and were shown to induce mobilization of bone marrow-derived cells, including circulating endothelial progenitor cells that colonize the viable tumor rim and contribute to tumor regrowth^6^,^24^. Collectively our results and the published data demonstrate that a robust pro-angiogenic response is induced by VDA treatment that contributes to tumor regrowth. In addition, to pro-angiogenic contribution these growth factors (HIF-1α, HGF, VEGF, and TGF-β) are known to induce and sustain EMT in tumors^25^–^27^ conferring invasiveness and resistance to drug treatments^28^,^29^.

The most striking finding of our study was the immediate and widespread EMT in virtually all surviving tumor cells in the periphery. The speed and the extent of this transition were totally unexpected. Although EMT has been implicated as a major mechanism for the development of drug resistance^9^, findings similar to ours have not been demonstrated previously. This may be related to the majority of published studies being conducted in vitro. In cell culture EMT induction in response to drug treatment or to growth factor stimulation occurs over a much longer period ranging from a few days to several weeks^26^,^30^. In addition, it has also not been seen in clinical samples as these tumor samples are not collected immediately after treatment. Recent publications reported increased mesenchymal morphology in recurrent tumors following longer treatment regimens. One such study reported that residual breast tumor biopsies exhibited both stem cell and mesenchymal features after neoadjuvant hormone or chemotherapy treatment^10^. Another study used oxaliplatin treatment on an orthotopic nude mouse model of human hepatocellular carcinoma, and demonstrated significantly increased metastasis and molecular changes consistent with EMT^31^. In both these studies tumor tissues were examined several days after completion of treatment. By this time tumors that were not completely destroyed are likely to have reestablished, and as shown in our studies, the majority of cells may have reverted to an epithelial state. Our study provides compelling histological evidence of EMT occurring following in vivo treatment. Within 1 h of treatment dramatic changes in four key EMT markers were observed; E-cadherin levels decreased dramatically, β-catenin was redistributed to cytoplasm and nucleus, while ZEB1 and vimentin levels increased dramatically. Nuclear localization of β-catenin indicates the activation of β-catenin-dependent Wnt signaling and the β-catenin/TCF transcriptional target genes that are associated with tumor invasion and metastasis^32^. An inverse correlation between ZEB1 and E-cadherin has been reported in clinical and experimental studies and this relationship is a prognostic indicator of poor survival and resistance to several drugs^9^. ZEB1 has also been shown to downregulate several other polarity proteins^33^. Clarhaut et al.^34^ demonstrated that silencing of ZEB1 resulted in upregulation of E-cadherin and increased drug sensitivity. ZEB1 has been associated with aggressive behavior of colorectal tumors and uterine cancers. In another study, upregulation of ZEB1 and the loss of E-Cadherin correlate with resistance to gefitinib (an epidermal growth factor receptor inhibitor), and with a poor prognosis^35^.

The most likely reason for the widespread EMT observed immediately following treatment is to protect the cells at the injury site by two mechanisms: by preventing proliferation and inhibiting apoptosis. Our previous findings demonstrated that both of these pathways were significantly impacted in the first 24 h of OXi4503 treatment within the remaining viable rim^5^ and support this hypothesis. Furthermore it has been postulated that EMT enables tumors to escape from apoptosis and senescence signals by adopting embryonic signaling pathways^36^,^37^. Temporal changes in ZEB1 levels, that very closely reflect our findings were reported by Bui et al.^38^ in a hypoxia-induced stroke injury model. In that study ZEB1 was shown to induce a pro-survival response by the transcriptional repression of pro-apoptotic factors and upregulation of pro-survival factors. The reduced apoptosis seen after Oxi4503 treatment in our earlier study may be due to a similar pro-survival function of ZEB1.

The very rapid onset of EMT following treatment suggests posttranscriptional regulation. It is well established that HIF-1α is stabilized by hypoxia at the protein level^39^. The protein levels of vimentin and ZEB1 in our study also increased far too quickly to be solely attributed to transcriptional upregulation initially. Recent studies have shown that regulation of protein expression also occurs at the translational level where key driver mRNAs are present but prevented from being translated by either miRNAs or inhibitory proteins^40^. This type of regulation is very important as it enables the cell to respond very fast to environmental changes and to produce key factors without the need for transcription. The rapid decrease in E-cadherin within the residual tumor is most likely the result of proteolysis rather than transcriptional inhibition. The increased ZEB1 levels also ensure transcriptional inhibition of E-cadherin further stabilizing the mesenchymal morphology of the residual tumor. ZEB1 is known to be regulated by a double negative feed forward loop involving the miRNA 200 family^30^,^41^. In ZEB1 the translational regulation may be achieved through association with miRNAs or other inhibitors preventing ribosomal binding of the ZEB1 mRNA. Vimentin, the other protein which also increased immediately following treatment, is a mesenchymal filament protein and is thought to have important functions in morphological reorganization of cells and cell motility hence may be associated with metastasis^42^.

EMT is a complex process and the mechanisms leading to its induction are not clear. In cell cultures EMT is usually induced by growth factors including HGF, VEGF, and most commonly TGF-β, which is widely considered to be the upstream driver of EMT^25^. In our study these growth factors took several hours after treatment to upregulate while TGF-β did not increase significantly until day five. By that time the EMT process was already reversing in the majority of tumor cells. HIF-1α and other transcription factors that upregulate immediately after treatment, as well as other proteins regulated at the translational level due to ensued hypoxia and injury, are likely to be responsible for the early EMT in drug treatment. However, TGF-β may be responsible for maintaining EMT morphology on an increasing fraction of tumor cells that disseminate and give rise to metastasis^25^. Tumor cells with mesenchymal morphology, in addition to being drug resistant, are known to be motile and invasive^9^. They are believed to give rise to metastasis by reverting to epithelial morphology through MET at distant metastatic sites^43^. While MET is implicated in the development of new metastases it has not been readily observed histologically in vivo. Two recent publications demonstrated that mesenchymal tumors induced by ectopic expression of EMT inducing transcription factors could only establish metastases if the expression of these transcription factors was reversed^44^,^45^. Our findings indicate that the majority of surviving cells are in the process of reverting to epithelial morphology at the site of the original tumor within 5 days posttreatment while they still retain significant mesenchymal features.

We also demonstrated robust EMT in response to other treatment modalities in the mouse CRCLM model, and in an orthotopic mouse breast tumor model. Collectively these results suggest that fast and widespread EMT is most likely a general response to hypoxic stress and tissue injury.

In conclusion our study suggests a central role of EMT in therapeutic failures and the likely involvement of translational regulation in drug induced EMT in vivo. In vitro studies may not dependably reflect in vivo changes in response to treatment because in vivo many factors contribute through autocrine and paracrine pathways to determine the final outcome. We identified a timeframe in therapy treatment that should be used to unravel important EMT mechanisms contributing to resistance and metastasis. The observation that all surviving tumor cells undergo EMT holds the promise that the recently described drugs which target cells with EMT/stem cell morphology such as salinomycin^46^ in combination with VDAs or other treatments including antiangiogenic agents may lead to more effective therapies.

## Supporting Information

Figure S1 Changes in caspase 3 staining of CRCLM tumors following OXi4503 treatment. Mice with liver metastases were treated with a single IP dose of OXi4503 (100 mg/kg) at 16 days post tumor induction. Tissues were collected at indicated times following OXi4503 treatment. First column, black bar = 250 μm, second column, black bar = 50 μm, third column = magnified inset of second column. L, liver; T, tumor; N, treatment induced necrosis. Double black lines in second column panels outline the live tumor rim after treatment. Red arrows in second and third panels of the second column indicate the viable tumor rim at 1 and 24 h after treatment. Positive caspase 3 staining (brown staining) indicating apoptotic cells or cell fragments. Double arrows in insets (column 3) indicate apoptotic cells in the necrotic area and within the viable tumor.

Figure S2 Temporal changes in VEGF and AT1R expression following OXi4503 treatment determined by immuno histochemistry. Tissues were collected at indicated times following OXi4503 treatment. Expression (brown staining) was detected using the appropriate antibodies. Black bar = 200 μm; L, liver; T, tumor; N, treatment induced necrosis. Single red arrows indicate that tumor cells within the viable rim at 1 and 24 h show increased VEGF and AT1R staining. Black arrows indicate areas of infiltrating cells positive for VEGF or AT1R.

Figure S3 Temporal changes in proangionenic growth factor concentration in liver lysates and serum following OXi4503 treatment. Tissues were collected at indicated times following OXi4503 treatment. Growth factor concentration was determined in lysates by capture ELISA assays using commercially available kits (R&D Duo Kits) ▪, control tumor bearing mice; □, Oxi4503 treated tumor bearing mice; □ naive mice (no tumor induction) The data are mean values _SEM (*n* = 10) First row, temporal changes in growth factors in liver lysates of treated animals were not significantly different from controls. Liver lysates from tumor bearing animals express significantly higher levels of all 3 growth factors compared to naive mice, HGF **P* < 0.005, TGF-β **P* < 0.01 and VEGF, **P* < 0.05. Second row, temporal changes in growth fac- compared to untreated control. TGF-β treated at 24 h **P* < 0.002 and **P* < 0.0009 at 5 days following OXi4503 treatment respectively compared to untreated controls. Sera from tumor bearing animals express significantly higher levels of TGF-β than sera from naive animals, **P* < 0.03.

Figure S4 Changes in EMT markers following OXi4503 treatment of tumor metastases. Mice with liver metastases were treated with a single IP dose of OXi4503 (100 mg/ kg) at 16 days post tumor induction. Tissues were collected at 1, 24 h and 5 days following OXi4503 treatment. Formalin fixed control and treated tumor sections in columns 1–4 were stained with antibodies to E-cadherin, β-catenin, vimentin and ZEB1 respectively. Positive expression is detected by the brown staining. Red arrows indicate area of the viable tumor rim at 1 and 24 h after treatment. Black bar = 250 μm. L, liver; T, tumor; N, treatment induced necrosis.

Table S1 List of antibodies and conditions used.
