# Supplementary figures and images for: Treatment with the vascular disruptive agent OXi4503 induces an immediate and widespread epithelial to mesenchymal transition in the surviving tumor
Source: Cancer Med. 2013 Aug 18;2(5):595–610. doi: 10.1002/cam4.109 (PMC3892792; doi:10.1002/cam4.109)

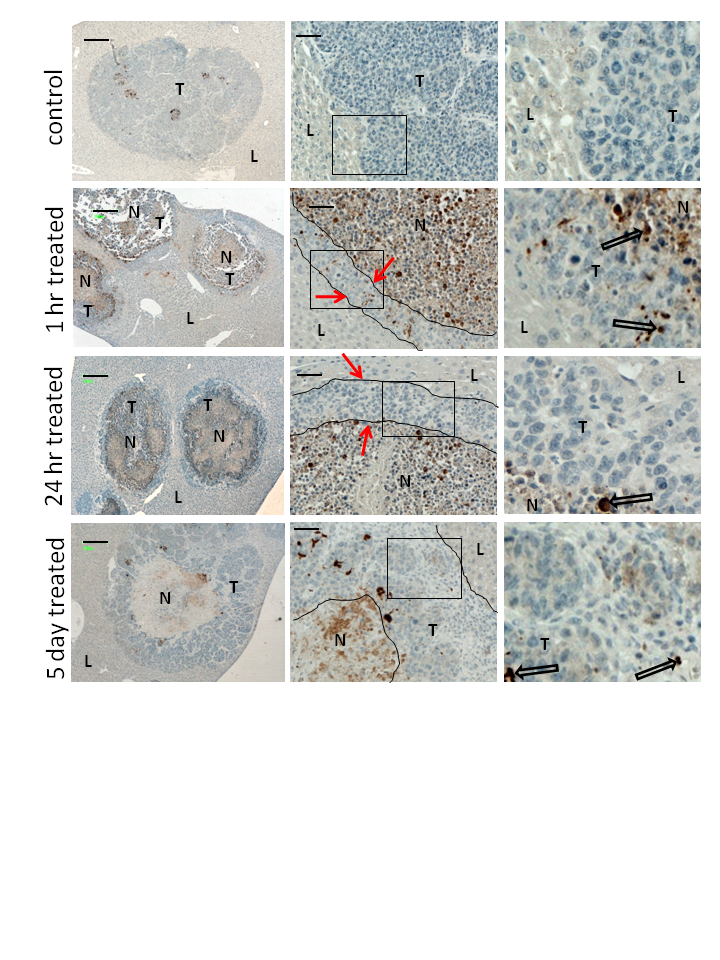

Supplement: Figure S1 — Changes in caspase 3 staining of CRCLM tumors following OXi4503 treatment. Mice with liver metastases were treated with a single IP dose of OXi4503 (100 mg/kg) at 16 days post tumor induction. Tissues were collected at indicated times following OXi4503 treatment. First column, black bar = 250 μm, second column, black bar = 50 μm, third column = magnified inset of second column. L, liver; T, tumor; N, treatment induced necrosis. Double black lines in second column panels outline the live tumor rim after treatment. Red arrows in second and third panels of the second column indicate the viable tumor rim at 1 and 24 h after treatment. Positive caspase 3 staining (brown staining) indicating apoptotic cells or cell fragments. Double arrows in insets (column 3) indicate apoptotic cells in the necrotic area and within the viable tumor. [file cam40002-0595-sd1.tif]

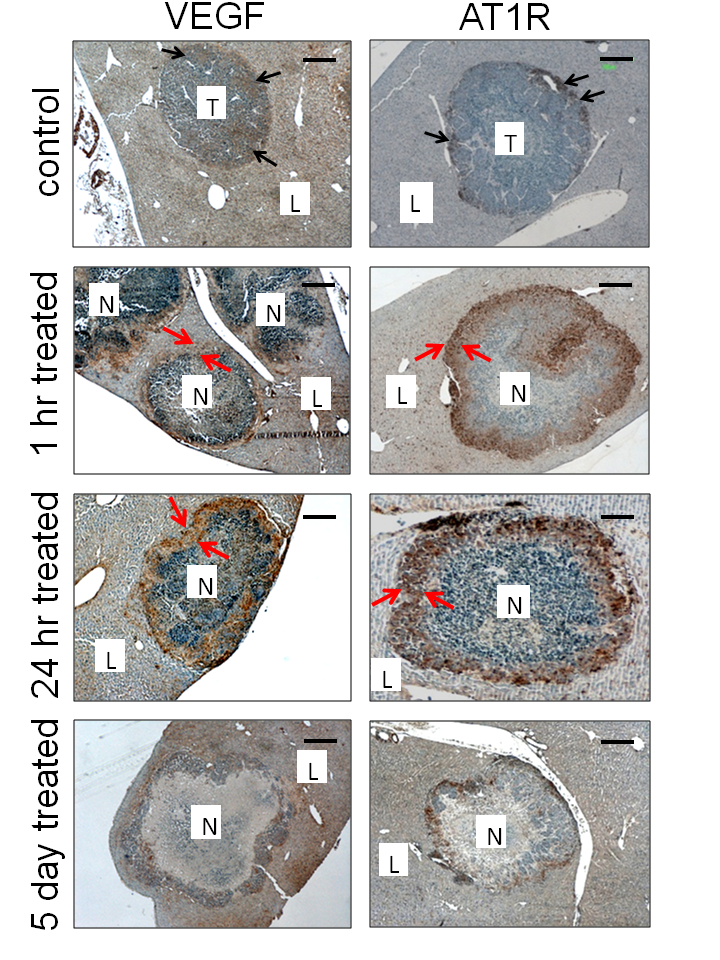

Supplement: Figure S2 — Temporal changes in VEGF and AT1R expression following OXi4503 treatment determined by immuno histochemistry. Tissues were collected at indicated times following OXi4503 treatment. Expression (brown staining) was detected using the appropriate antibodies. Black bar = 200 μm; L, liver; T, tumor; N, treatment induced necrosis. Single red arrows indicate that tumor cells within the viable rim at 1 and 24 h show increased VEGF and AT1R staining. Black arrows indicate areas of infiltrating cells positive for VEGF or AT1R. [file cam40002-0595-sd2.tif]

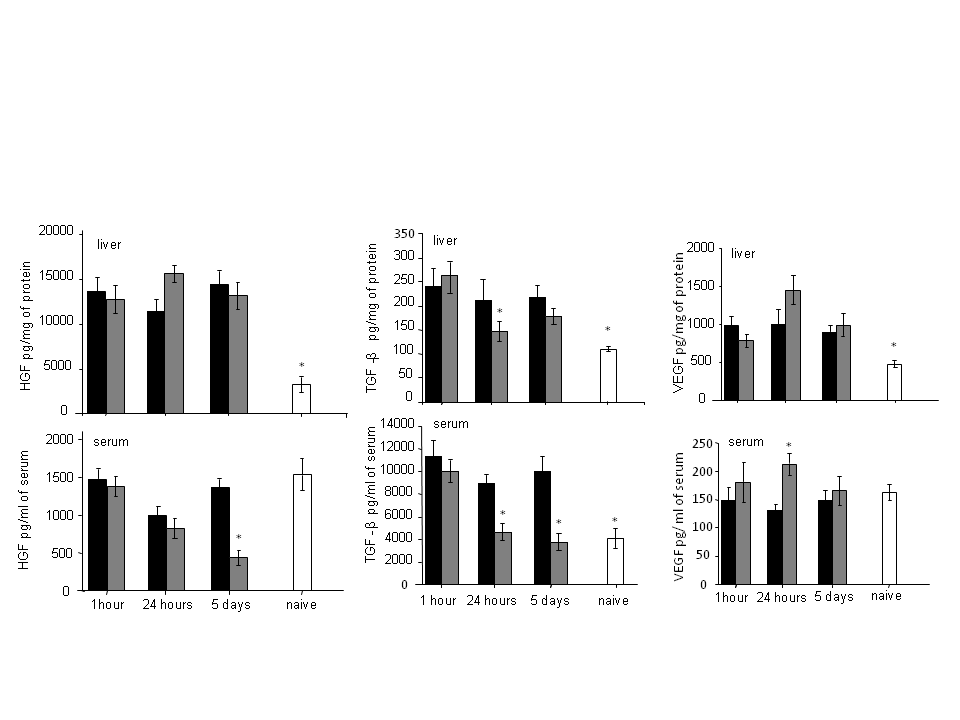

Supplement: Figure S3 — Temporal changes in proangionenic growth factor concentration in liver lysates and serum following OXi4503 treatment. Tissues were collected at indicated times following OXi4503 treatment. Growth factor concentration was determined in lysates by capture ELISA assays using commercially available kits (R&D Duo Kits) ▪, control tumor bearing mice; □, Oxi4503 treated tumor bearing mice; □ naive mice (no tumor induction) The data are mean values _SEM (n = 10) First row, temporal changes in growth factors in liver lysates of treated animals were not significantly different from controls. Liver lysates from tumor bearing animals express significantly higher levels of all 3 growth factors compared to naive mice, HGF *P < 0.005, TGF-β *P < 0.01 and VEGF, *P < 0.05. Second row, temporal changes in growth fac- compared to untreated control. TGF-β treated at 24 h *P < 0.002 and *P < 0.0009 at 5 days following OXi4503 treatment respectively compared to untreated controls. Sera from tumor bearing animals express significantly higher levels of TGF-β than sera from naive animals, *P < 0.03. [file cam40002-0595-sd3.tif]

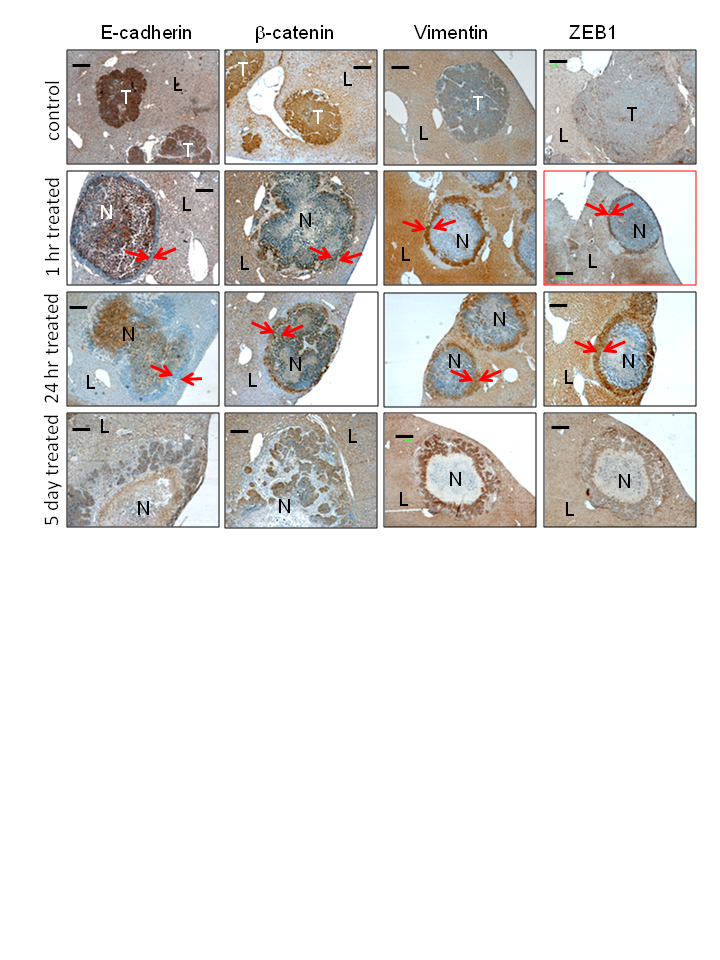

Supplement: Figure S4 — Changes in EMT markers following OXi4503 treatment of tumor metastases. Mice with liver metastases were treated with a single IP dose of OXi4503 (100 mg/ kg) at 16 days post tumor induction. Tissues were collected at 1, 24 h and 5 days following OXi4503 treatment. Formalin fixed control and treated tumor sections in columns 1–4 were stained with antibodies to E-cadherin, β-catenin, vimentin and ZEB1 respectively. Positive expression is detected by the brown staining. Red arrows indicate area of the viable tumor rim at 1 and 24 h after treatment. Black bar = 250 μm. L, liver; T, tumor; N, treatment induced necrosis. [file cam40002-0595-sd4.tif]
