# Supplementary material for: Treatment with the vascular disruptive agent OXi4503 induces an immediate and widespread epithelial to mesenchymal transition in the surviving tumor
Source: Cancer Med. 2013 Aug 18;2(5):595–610. doi: 10.1002/cam4.109 (PMC3892792; doi:10.1002/cam4.109)
Supplement: Table S1 — List of antibodies and conditions used. [file cam40002-0595-sd5.doc]

Supplemental Table 1 **List of antibodies and conditions used.**

| **Target protein** | **Company** | **Antibody** | **Dilution** | **Conc. (μg/ml)** | **Antigen retrieval** |
| --- | --- | --- | --- | --- | --- |
| β-catenin | Santa Cruz | Rat anti-mouse monoclonal sc-7199 | 1:300 | 0.67 | Tris |
| E-cadherin | Santa Cruz | Rabbit anti-mouse sc-7870 | 1:500 | 0.4 | Citrate |
| Vimentin | Santa Cruz | Rabbit anti-mouse sc-5568 | 1:300 | 0.67 | Tris |
| ZEB1 | Santa Cruz | Rabbit anti-mouse sc-25388 | 1:200 | 1 | Tris |
| AT1R | Santa Cruz | Rabbit anti-mouse  sc-1173 | 1:300 | 0.67 | N/A |
| VEGF | CalBiochem***,*** | Rabbit anti-mouse PC315 | 1:400 | 2.5 | Proteinase K |
| HIF1-α | Chemicon | Rabbit anti-mouse AB 3883 | 1:100 | 60 | Tris |
